# Targeting inaccuracy caused by mechanical distortion of the Leksell stereotactic frame during fixation

**DOI:** 10.1002/acm2.12576

**Published:** 2019-04-04

**Authors:** Cécile Renier, Nicolas Massager

**Affiliations:** ^1^ Department of Radiotherapy CHU‐UCLouvain Namur Belgium; ^2^ Department of Neurosurgery University Hospital Tivoli La Louvière Belgium

**Keywords:** inaccuracy, Leksell stereotactic frame, mechanical distortion, radiosurgical procedures, stereotactic surgery

## Abstract

**Background:**

The stereotactic frame represents the mainstay of accuracy for targeting in stereotactic procedures. Any distortion of the frame may induce a significant source of error for the stereotactic coordinates.

**Objective:**

To analyze the sources of distortion of the Leksell frame G induced by fixation to the patient's head and to evaluate the clinical impact of frame distortion on the accuracy of targeting in stereotactic procedures.

**Methods:**

We analyzed the torques exerted on the fixation screws after frame placement in a series of patients treated stereotactically by an experienced team. We studied the risk for frame bending in an experimental model of stereotactic frame fixation, with increasing torque of fixation screws in a homogeneous and heterogeneous distribution of torques between the four screws. We assessed the impact of expanding dimensions of bending of the Leksell frame both on surgeries utilizing the stereotactic frame, and on radiosurgical procedures with the Gamma Knife.

**Results:**

Frames were fixed clinically at a range of torques of 0.147–0.522 Nm (mean = 0.348 Nm). The torques did not vary significantly with time. Heterogeneity between the two opposite pairs of screws is often limited, but can reach 96.3%. Distortion of the frame may occur even at minimal levels of torque. Heterogeneity between the two opposite pairs of screws will significantly raise the amount of frame distortion. We found a direct correlation between measures of the frame distortion and extend of the deviation from the stereotactic target in clinical models of stereotactic procedures.

**Conclusion:**

Stereotactic frames were subjected to distortion due to the torque used for frame fixation. The risk of distortion increased with the torque used and the heterogeneity between the torques of the fixation screws. Distortion of the frame was a significant source of inaccuracy of targeting for stereotactic procedures in clinical practice.

## 1 INTRODUCTION

Efficient stereotactic surgery and Gamma Knife radiosurgery procedures require a high spatial accuracy. This accuracy is achieved by the use of a stereotactic frame, which is firmly fixed to the patient's head. The stereotactic frame with a localizer fixed to it allows the registration of the patient's anatomy for the three‐dimensional coordinate system defined by the frame for accurate placement of surgical instruments or for targeting radiosurgical irradiation. To obtain high accuracy during this stereotactic procedure, the stability of all elements of the system is crucial. Among other things, the mechanical stability of the stereotactic frame is a critical issue since it represents the central element for the definition of stereotactic coordinates and the support for targeting.^1^

Treuer et al.^2^ published the results of a study on the influence of stereotactic frame distortions on targeting accuracy where they showed that in a series of patients undergoing a stereotactic procedure with a stereotactic frame, a mean frame bending of 0.74 mm and maximal bending of 1.30 mm occurred. They advocated that a too firm attachment of a stereotactic head frame to the patient's skull may cause distortion of the frame in clinical practice. They further suggested that the alloy Al‐Cu‐Mg used as components of the conventional frames allows such distortion and that the use of frames in ceramics could resolve the problem of frame bending.^2^ It remains to be known if the distortion of the stereotactic frame will affect the accuracy of stereotactic targeting. Current stereotactic procedures use CT and/or MR imaging acquired after placement of a CT‐ or MR‐indicator box on the frame. What error frame bending will induce on the stereotactic coordinates obtained with this imaging is difficult to estimate. Treuer et al.^2^ assessed that with a CT‐ or MR‐localizer with lateral plates fixed with a plate at the top of the localizer, frame distortion will have no significant clinical impact on the stereotactic accuracy of the system. They also asserted that frame bending will have no significant clinical effect in radiosurgery, although noted that frame distortions may lead to some minimal errors in stereotactic localization and consequently could limit the target point accuracy in radiosurgery, which contradicts the earlier assertion.

**Figure 1:**
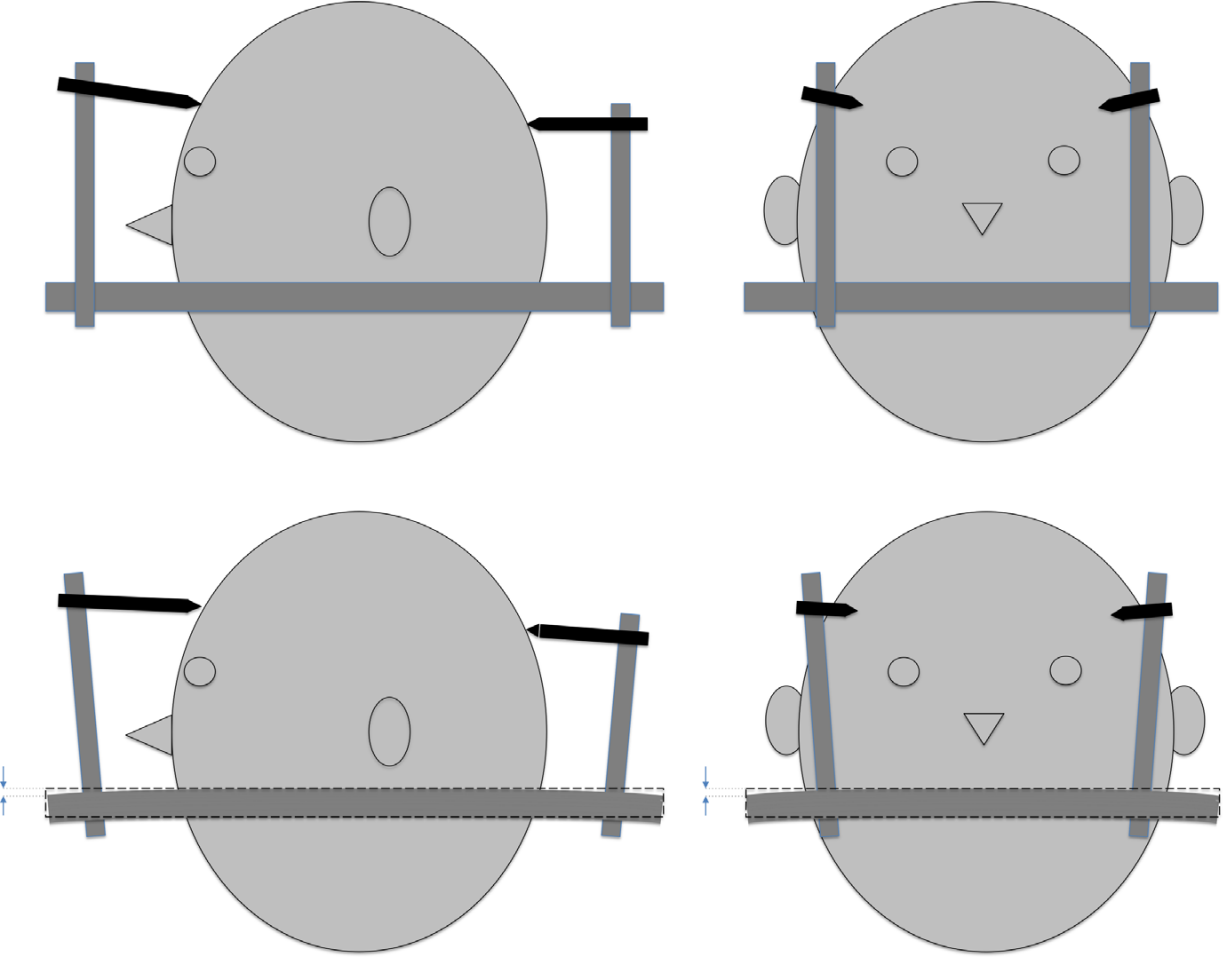
**Illustration of the distortion of the Leksell stereotactic frame due to patient fixation. Superior images: Sagittal and coronal view without distortion. Inferior images: Sagittal and coronal view with distortion.**

The aim of our study was to analyze in which circumstances the Leksell stereotactic frame G (Elekta Instruments AB, Stockholm, Sweden) could be mechanically deformed during a stereotactic procedure. The present work focused on the Leksell frame distortions that could be induced by the torques exerted during fixation of the frame on the patient's head (Fig. 1). We analyzed Leksell frames mounted with conventional posts and conventional screws, as suggested in the Instructions for Use.^3^ Also, the genuine clinical effect of distortion of the stereotactic frame stays obscure: Does frame bending truly incite a problem in stereotactic focusing for surgeries, for example, DBS surgical procedure and frame‐based biopsy, and for Gamma Knife radiosurgical treatment? Different referential systems are fixed on the stereotactic frame for these techniques, and the fixation of different stereotactic instruments (stereotactic arc for surgical procedures, frame adapter for Gamma Knife) to the frame could likely impact in various ways the outcomes of frame distortion on the accuracy of targeting.

## 2 MATERIALS AND METHODS

This study was approved by the local Institutional Review Board by the Ethical Committee of our institution (ref. P2015/207). Informed consent was obtained in all patients included in this study.

Our study consists of three different parts. In the first part, we analyzed the clinical expertise of routine procedures of frame fixation by an experienced team and defined the normal range of torques exerted on the Leksell frame by fixation to the patient's head. In the second part, we study the risk of frame distortions created by the use of different levels of torque exerted on the frame with the four fixation screws. In the third part of this study, we studied the clinical impact of frame bending on two different stereotactic applications of the Leksell frame G.

### 2.A Clinical experience of frame fixation

Our team had a 20‐year experience with more than 4000 applications of stereotactic head frame in a routine clinical setting of stereotactic and functional neurosurgery and Gamma Knife radiosurgery. For this clinical study, we used a high‐precision digital torque screwdriver (E.S404, Facom S.A.S.^®^, Morangis, France), self‐calibrated, which allows measurements of torque with an accuracy of 3%. Using this screwdriver, we measured the torque exerted individually by the four screws at the end of the frame fixation procedure in 75 patients. We made the same measurements at the end of the stereotactic procedure, before frame removal. The measurements were expressed in Newton‐meter (Nm); conversion to inch‐pound is: 0.1 Nm (Newton‐meter) = 0.885 inch‐pound; 4 inch‐pounds = 0.452 Nm. The torques T1, T2, T3, and T4 (recorded at end of frame fixation) and T5, T6, T7, and T8 (recorded at end of the stereotactic procedure, before frame removal) were measured as the torque of the left anterior, right anterior, right posterior, and left posterior screw, respectively.

### 2.B Frame distortion model

**Figure 2:**
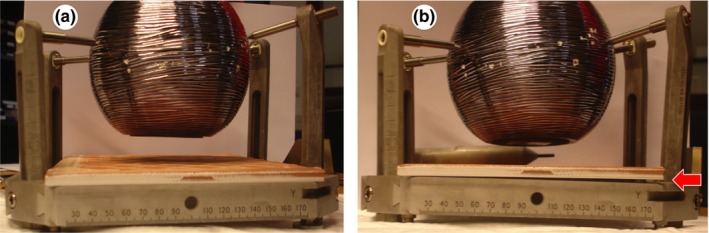
Image of distortion of the frame by asymmetric torques exerted during frame attachment. Left image: No torque exerted and no frame distortion. Right image: asymmetric torques (T1 = T3 = 0.25 Nm, T2 = T4 = 0.8 Nm), frame distortion with a gap of 2.7 mm (red arrow).

We developed an experimental model to analyze the distortion of the Leksell stereotactic frame G induced by the torques exerted by frame fixation. The model used a rigid sphere of 15 cm diameter. The Leksell frame G was tightened on this sphere with four fixation screws, as used clinically [Fig. 2(a)]. Frame bending was measured using a rigid flat panel of 17 × 22 cm dropped on both lateral sides of the superior base of the frame. Without frame distortion, the four corners of the panel were in contact with the upper surface of the lateral sides of the frame [Fig. 2(a)]. If distortion of the frame occurred, a gap was visible at one corner of the panel [Fig. 2(b), red arrow], and only on one lateral face of the frame. This gap was measured with 0.05 mm thick feeler gauges to evaluate the thickness by a multiple of 0.05 mm. The measurements of the gap induced by frame bending were performed by an independent observer.

#### 2.B.1 Homogeneous torque exerted

In the first part of this experimental study, we checked the influence of an increase in levels of torques homogeneously exerted on the four screws on frame bending. For this experiment, we fixed the frame with four screws tightened to the same torque, according to a protocol for incrementing to different torque levels from 0 to 0.8 Nm in steps of 0.1 Nm. For each level of torque, we measured frame bending for each of the seven Leksell frames G analyzed.

#### 2.B.2 Heterogeneous torque exerted

**Figure 3:**
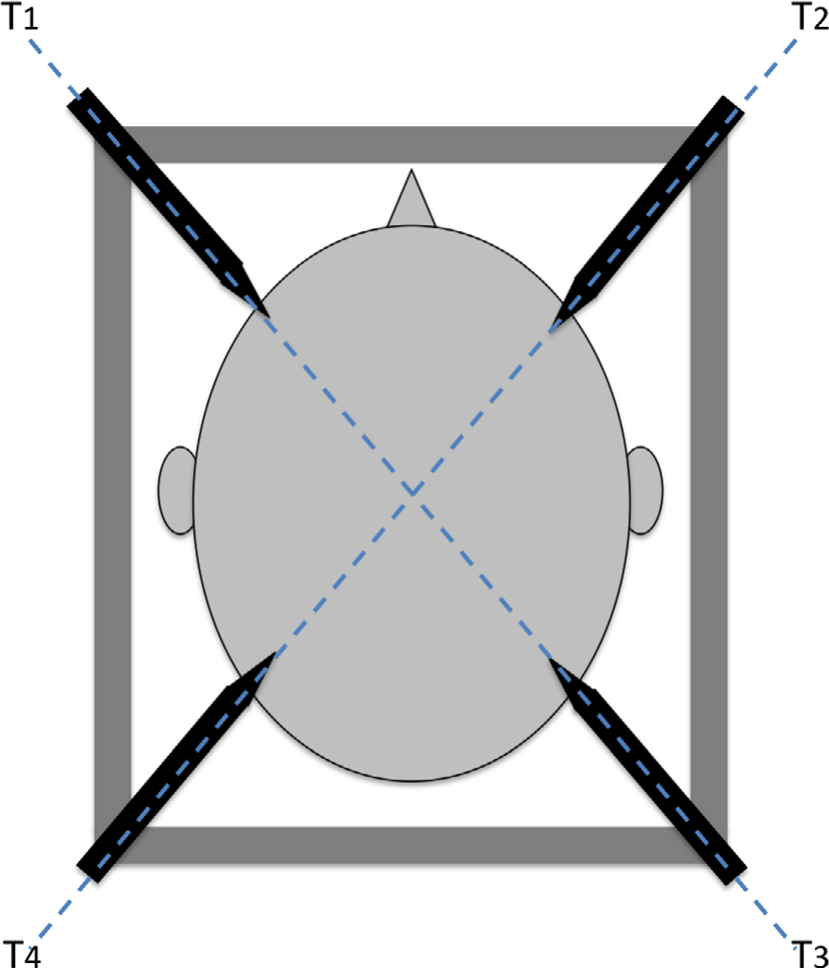
Visualization of the torques T1, T2, T3, and T4 exerted to the frame by head fixation.

In the second part of this study, we analyzed frame distortion caused by frame fixation when torques were exerted at different levels between the 2 sets of 2 diagonally opposite screws, as shown in Fig. 3. T1 and T3 were set at a similar level of torque, T2 and T4 were also set at a similar level of torque, and different levels of torques were set between T1 and T2. Although we found from our clinical experience that only one screw set at a significantly different torque than the three others could alter coplanar properties of the frame, distortion of the frame seems much more important when the two couples of diagonally opposite screws are set at different levels of torque. From this experience, we followed a protocol of increasing torque levels for both couples, as listed in Table 3, columns 1 and 2. Frame bending was measured with the same procedure as already described above.

### 2.C. Clinical impact of frame distortion on surgery and radiosurgery targeting accuracy

The clinical consequences of frame bending have been studied on two different stereotactic applications of the Leksell Frame G.

#### 2.C.1 Surgical procedures

**Figure 4:**
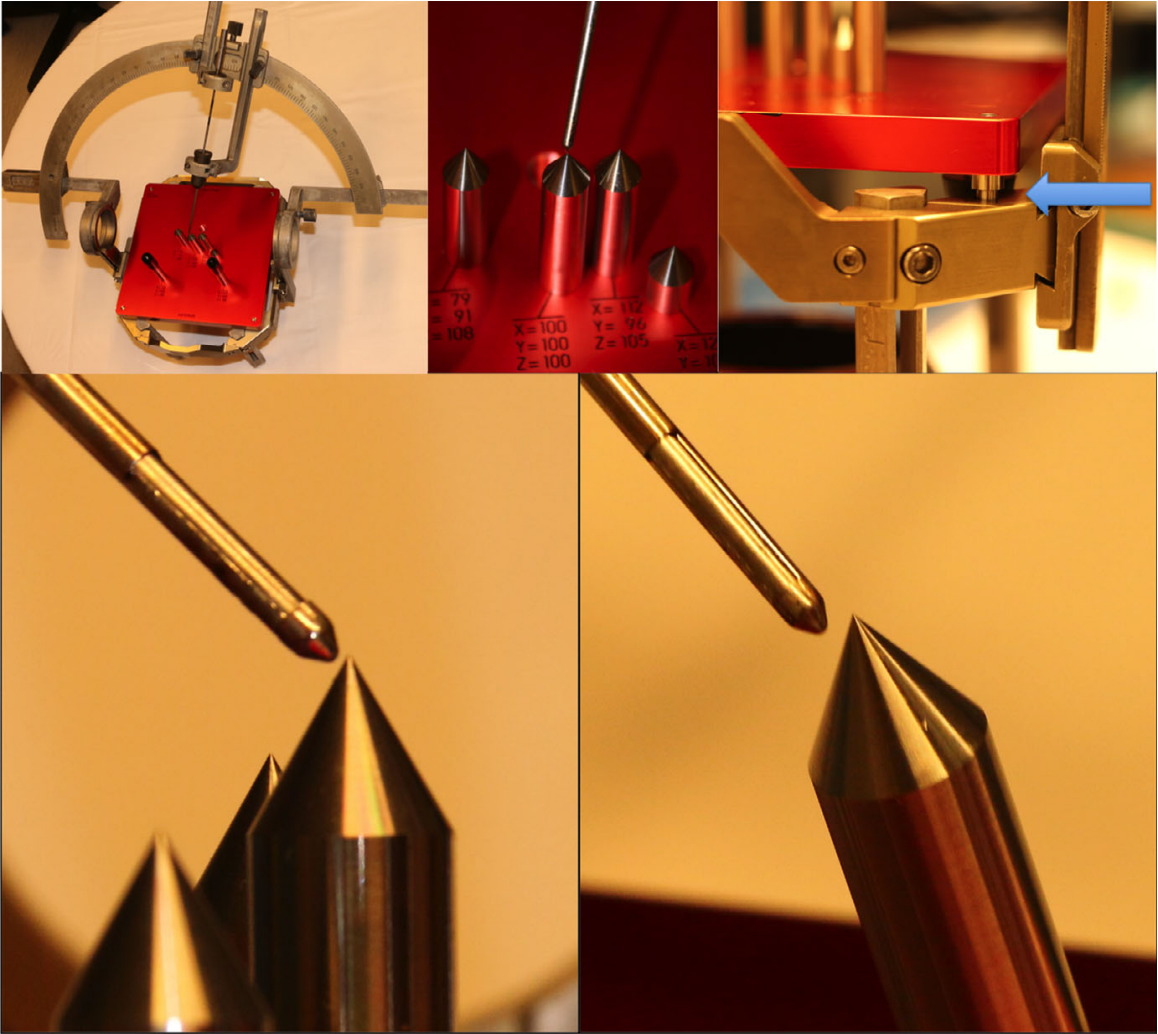
Use of the Target Simulator of Elekta^®^ to study the effect of frame distortion on targeting of surgical procedures using the stereotactic arch applied to the frame. Lower left: Using a low torque, the biopsy needle reached the stereotactic target. Lower right: Using a high torque in order to create a distortion of the frame inaccuracy of targeting of the biopsy needle.

This examination would ponder the impact of frame distortion on targeting in 60 surgeries performed with the Leksell stereotactic arch attached to the frame. For this reason, we have utilized the Target Simulator of Elekta^®^ (Elekta Instruments, Sweden). We connected the Target Simulator on a stereotactic frame not stressed by any significant torque. We settled the stereotactic arch on the frame and we utilized a Sedan biopsy needle of 2.5 mm diameter to reach the target (Fig. 4, upper left and center). We put the stereotactic coordinates on the frame. We measured the precision of targeting provided by the needle. Then, we mounted on the reverse part of the frame a rigid sphere of 15 cm diameter tightened with four fixation screws, and we applied different conditions of torque in order to create six values of frame bending: 0.5, 1.0, 1.5, 2.0, 2.5, and 3.0 mm (Fig. 4, upper right). We measured the modifications of accuracy of targeting of the biopsy needle in relation to the target provided by the Target Simulator (Fig. 4, lower left and right) by adjustments of the x, y, and z coordinates on the stereotactic arch to make the needle and the target coincide.

#### 2.C.2 Gamma Knife procedures

This experimental study focused on the influence of distortion of the Leksell frame on the radiation targeting of the Leksell Gamma Knife Icon^®^ (Elekta Instruments, Sweden). We placed the Leksell frame on the head of an anthropomorphic phantom and we applied different conditions of torque in order to create increasing distortions of the stereotactic frame. A series of 30 measurements were performed when the frame was not distorted and with five levels of frame distortions at 0.5, 0.85, 1.0, 1.5, and 2.0 mm: we run each experiment multiple times, and Table 4 represents the average of these measurements. We evaluated the targeting inaccuracy induced by distortion of the frame using the following method: we compared the stereotactic coordinates of a target located in the head based on two different stereotactic CT scans. The first one was the conventional stereotactic 1‐mm 3D CT scan acquired after having placed the CT Indicator box of Elekta^®^ (Elekta Instruments, Sweden) on the frame, as used in a clinical routine. The location of the fiducial marker sets on the tomographic images was used by the Stereotactic Reference Definition program of Leksell GammaPlan 11.0 to define the stereotactic coordinates of tomographic images. The indicator box was docked on the frame; so that bending of the frame would unavoidably influence the stereotactic coordinates issued from the box. The second stereotactic CT scan was the stereotactic Cone Beam CT (CBCT) of the Gamma Knife Icon.^4^, ^5^ The CBCT based stereotactic localization was based on the registration between the CT and CBCT of the anthropomorphic phantom. The acquisition of stereotactic coordinates from the CBCT is independent of any CT indicator box and is directly provided by the Gamma Knife^®^ ICON^TM^ system once the head frame has been docked on the couch with the frame adapter. So, the CBCT‐related stereotactic coordinates are independent of frame bending. We analyzed the difference of stereotactic coordinates of the target provided by these two imaging methods with GammaPlan software (Elekta Instruments, Sweden). We co‐registered both data sets and obtained two sets of stereotactic coordinates of the target. The difference in stereotactic coordinates will represent the inaccuracy of targeting induced by frame distortion in real clinical conditions. The ICON procedure using CBCT as reference will give corrections of coordinates in the three directions and vectorial correction that must be applied to correct the inaccuracy of targeting.^4^, ^5^ In order to confirm the accuracy of the correction applied, we used gafchromic films to irradiate the target without and with the correction proposed by the Gamma Knife ICON.

## 3 RESULTS

### 3.A Clinical experience of frame fixation

The standard procedure of frame fixation recommended by Elekta^3^ and described by others^6^ was used. The results of these measurements are presented in Table 1. The mean torque exerted on the four screws by frame fixation was 0.348 Nm (3.08 inch‐pound) and SD was 0.8 Nm. The minimum torque used for secured fixation was 0.147 Nm, and the maximum torque was 0.522 Nm.

**Table 1:** Analysis of torques exerted for frame fixation in a series of 75 patients

Number of patients	75
Torque (T) after frame fixation	
Mean	0.348 Nm
Median	0.347 Nm
Min	0.147 Nm
Max	0.522 Nm
SD	0.072 Nm
T_{fixation}_/T_{removal}_	
Mean	0.98
Median	0.99
Min	0.73
Max	1.19
SD	0.11
Δ T of opposite screws (P1P3 vs P2P4)	
Mean	0.086 Nm
Median	0.068 Nm
Min	0 Nm
Max	0.325 Nm
SD	0.060 Nm
% mean	26.4%
% median	22.4%
% min	0%
% max	96.3%
% SD	19.5%

To evaluate the applied torque as a function of time, we measured four torques at T1 to T4 at the end of the frame fixation procedure, and again at the end of the stereotactic treatment several hours later, just before frame removal. We calculated the mean value of the four torques measured at each time point. The median ratio between the two values was 0.99 (SD 0.1), and ranged from 0.73 to 1.19.

We also studied the difference between the torques that were applied by the neurosurgeon during frame placement by measuring the difference in torque (∆T) between the two different couples of diagonally opposed screws, T1–T3 and T2–T4. We found a mean and a median difference of torques of 0.086 and 0.068 Nm (SD 0.066), respectively. The maximum ∆T observed was 0.325 Nm. In addition to measurements in absolute values, we analyzed the value of ∆T as a percentage of the mean values of the four torques applied. We observed a mean and median ∆T of 26.4% and 22.4% (SD 21.8%) respectively, with a maximum ∆T of 96.3%.

### 3.B Frame distortion model

We applied our experimental protocols of frame distortion with homogeneous torques and heterogeneous torques to 7 different Leksell frames G. All these frames were certified to be within industry standards for accuracy during the 6‐month maintenance provided by Elekta Instruments^®^, including new frames as well as frames with several years of use.

#### 3.B.1 Homogeneous torque exerted

**Figure 5:**
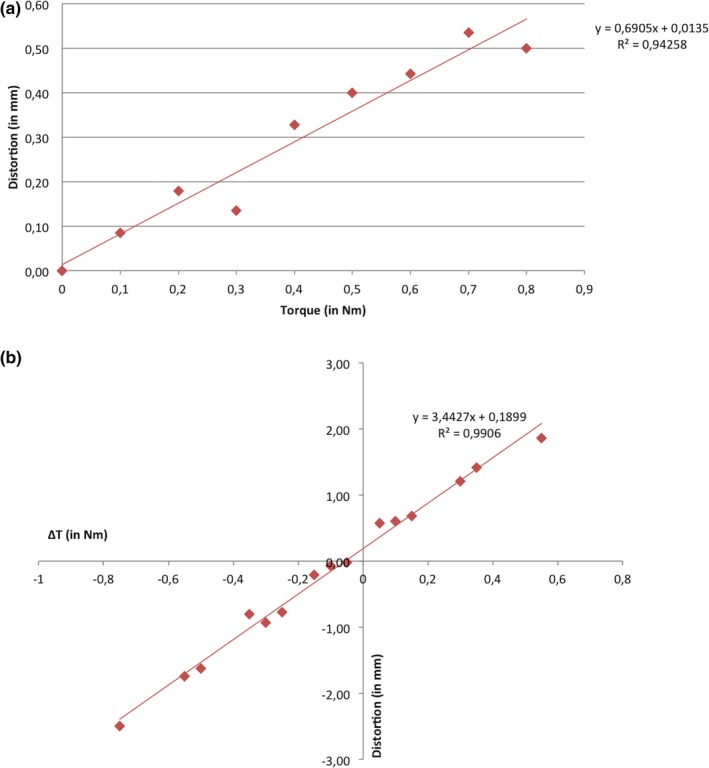
(a) Linear regression of the measurements of frame distortion induced by homogeneous torques used for frame fixation. (b) Linear regression of the measurements of frame distortion induced by heterogeneous torques used for frame fixation.

The results of the homogeneous torque exerted are presented in Table 2. Data were expressed as mean values for the measurements carried out on the seven frames for each torque level. Figure 5(a) shows the linear regression between the torque exerted on the frame and the mean frame distortion measured in the seven frames. The mean and maximum frame bending increased to 0.50 and 1.35 mm respectively when torques of 0.8 Nm were used. Some frames showed no bending under high torque while others showed bending at torques as low as 0.1 Nm. Since both old and new frames showed distortion equally, frame age does not appear to be a factor in its response to torque. The linear regression (Excel software, Microsoft) of these experimental data showed a correlation coefficient (r) of 0.9709; R^2^ = 0.94258. In the range of torques exerted routinely under clinical conditions, frames could have maximum distortions reaching 0.75 mm. However, the mean torque usually applied for frame attachment provided a frame bending in the range of 0.14–0.33 mm based on our measurements.

**Table 2:** Measurements of frame distortion induced by different conditions of torque exerted by the frame fixation posts: homogeneous torque applied

STUDY #1	Bending			
T1 = T2 = T3 = T4	Mean (mm)	Min (mm)	Max (mm)	SD
0	0	0	0	0
0.1	0.09	0	0.20	0.07
0.2	0.18	0.05	0.4	0.14
0.3	0.14	0	0.4	0.15
0.4	0.33	0.10	0.55	0.17
0.5	0.40	0	0.75	0.24
0.6	0.44	0	1.05	0.26
0.7	0.54	0	1.35	0.33
0.8	0.50	0	1.35	0.31

#### 3.B.2 Heterogeneous torque exerted

The results of the heterogeneous torque exerted are shown in Table 3. We found that the heterogeneity in the torques applied was associated with a marked increase in frame distortion. The difference between the torques (∆T) induced a distortion of the frame additional to the frame bending provided by equalized torques. The linear regression between frame bending and ∆T showed a correlation coefficient (r) of 0.9953; R^2^ = 0.9906 [Fig. 5(b)]. For the highest value of ∆T of our experimental protocol, 0.750 Nm, frames were distorted by mean and maximum values of 2.50 and 3.10 mm respectively. In our clinical daily practice, frame fixation to the patient's head has induced mean ∆T minimum of 0.1 Nm but maximal values that can reach 0.325 Nm. These levels of ∆T can produce distortion of the frame by more than 1 mm.

**Table 3:** Measurements of frame distortion induced by different conditions of torque exerted by the frame fixation posts: heterogeneous pressures applied

STUDY #2			DISTORTION (mm)			
T1 = T3 (Nm)	T2 = T4 (Nm)	ΔT (Nm)	Mean	Min	Max	SD
0.25	0	−0.25	−0.77	−1.2	−0.4	0.25
0.25	0.2	−0.05	−0.02	−0.7	0.87	0.49
0.25	0.4	0.15	0.69	0	1.85	0.61
0.25	0.6	0.35	1.41	0.45	2.6	0.73
0.25	0.8	0.55	1.86	0.9	2.7	0.72
0.5	0	−0.50	−1.62	−2.25	−1.05	0.39
0.5	0.2	−0.30	−0.93	−1.45	−0.37	0.42
0.5	0.4	−0.10	−0.07	−0.45	0.33	0.34
0.5	0.6	0.10	0.60	0.1	1.23	0.35
0.5	0.8	0.30	1.21	0.9	1.88	0.35
0.75	0	−0.75	−2.50	−3.1	−1.85	0.47
0.75	0.2	−0.55	−1.74	−2.45	−1.2	0.43
0.75	0.4	−0.35	−0.81	−1.2	−0.15	0.41
0.75	0.6	−0.15	−0.21	−0.95	0.45	0.51
0.75	0.8	0.05	0.58	−0.05	1.45	0.62

### 3.C Clinical impact of frame distortion on surgery and radiosurgery targeting accuracy

#### 3.C.1 Surgical procedures

**Figure 6:**
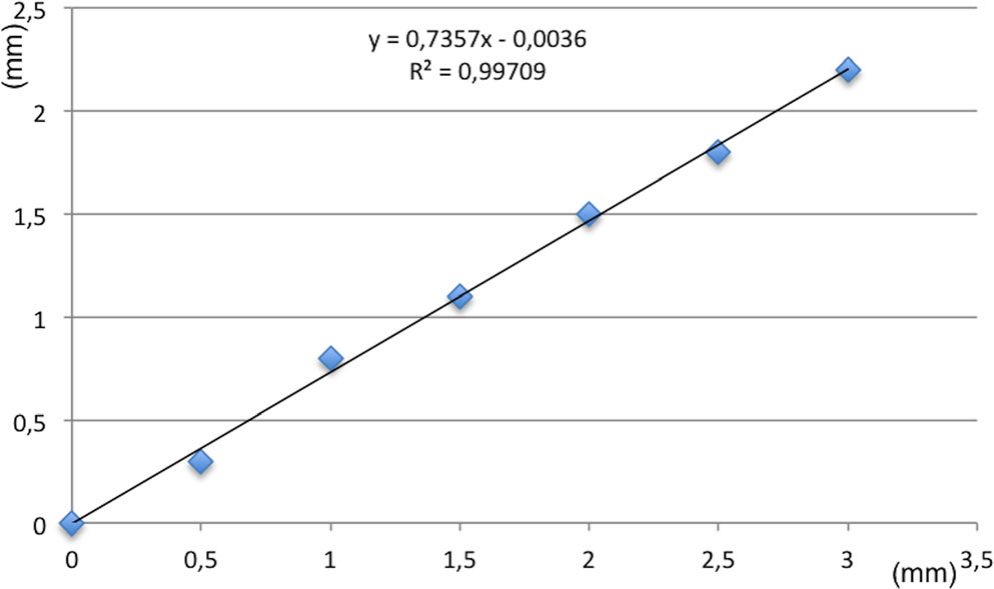
Graph showing the relation between frame distortion (*X*‐axis, in mm) and targeting inaccuracy (*Y*‐axis, in mm) with the Target Simulator model.

When the Target Simulator was applied to the stereotactic frame not strained by any significant torque, we observed that the needle reached the target with submillimetric precision. For the six values of frame bending 0.5, 1.0, 1.5, 2.0, 2.5, and 3.0 mm, we measured the distance of the tip of the stereotactic cannula from the target. The values recorded were 0.3, 0.8, 1.1, 1.5, 1.8, and 2.2 mm (Fig. 6). The trend line had an R^2^ of 0.99709. The equation of the trend line was y = 0.7357x + 0.036.

#### 3.C.2 Gamma Knife procedures

**Figure 7:**
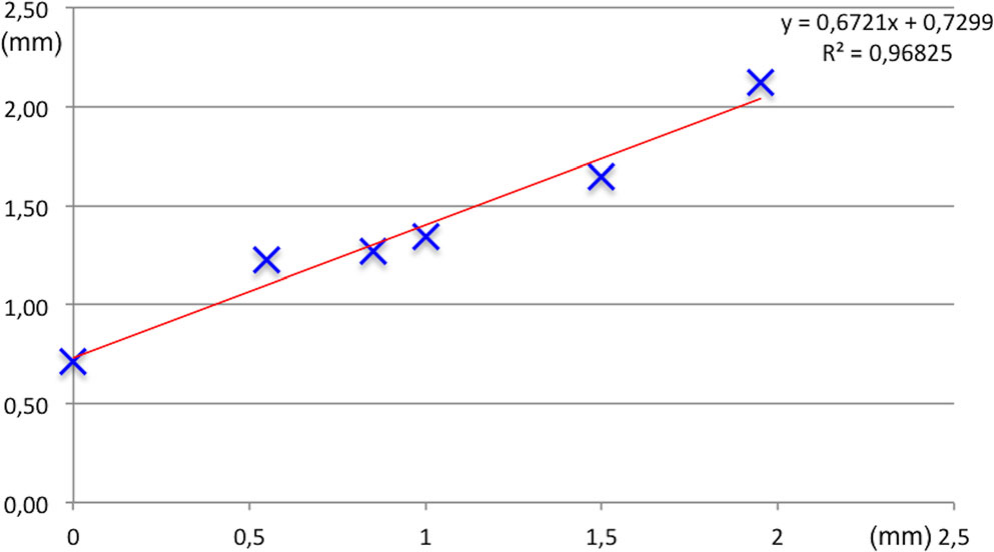
Graph showing the relation between frame distortion (*X*‐axis, in mm) and corrections of the stereotactic coordinates provided by the Cone Beam CT ‐related ICON procedure (*Y*‐axis, in mm).

The measurements performed when the frame was not distorted showed that the difference in stereotactic coordinates was a translation of −0.31 mm in *X*‐axis, −0.55 in *Y*‐axis, and −0.18 in *Z*‐axis. When increasing distortions were applied to the frame, the differences in stereotactic coordinates were increasing, as shown in Table 4. The vectorial deviation was 0.71 mm when the frame was not bent (physical inaccuracy of the frame) and increased to a maximum of 2.12 mm with increasing frame distortion. The trend line (Fig. 7) had an R^2^ of 0.96825. The equation of the trend line is y = 0.6721x + 0.7299.

**Table 4:** Corrections of the stereotactic coordinates provided by the CBCT‐related ICON procedure when different levels of frame distortion are applied

Distortion (mm)	*X*‐axis (mm)	*Y*‐axis (mm)	*Z*‐axis (mm)	Vectorial (mm)
0	−0.63	−0.31	−0.10	0.71
0.5	−1.21	−0.18	0.03	1.22
0.85	−1.24	−0.26	0.10	1.27
1.0	−1.33	−0.09	0.13	1.34
1.5	−1.63	−0.03	0.24	1.65
2.0	2.08	−0.02	0.41	2.12

**Figure 8:**
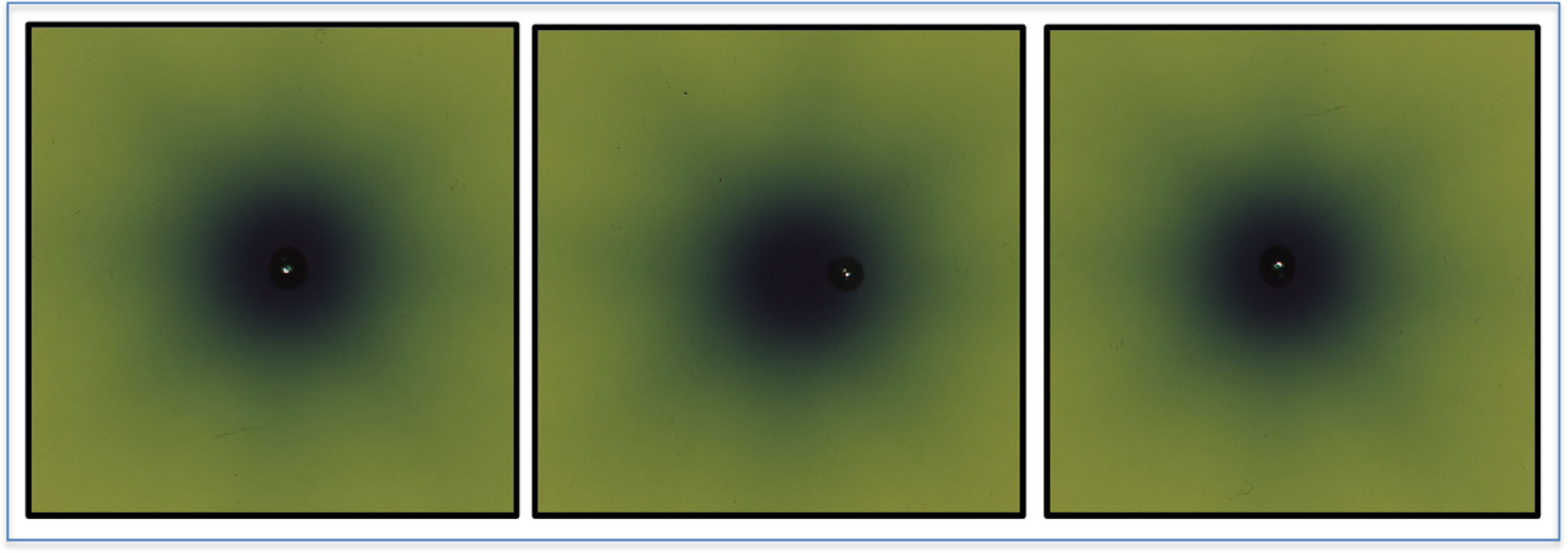
Gafchromic films after irradiation of the target when the stereotactic coordinates from the CT indicator box were used with a frame not distorted (left), when the stereotactic coordinates from the CT indicator box were used with a frame distorted by 3.0 mm (median), and when the stereotactic coordinates from the Cone Beam CT of the ICON procedure were used with a frame distorted by 3.0 mm (right). The target was reached with a high accuracy when the frame was not bent (left). With a frame distorted by 3.0 mm, the target was significantly shifted when stereotactic coordinates from the CT indicator box were used (median) and was reached with a high accuracy when the stereotactic coordinates from the Cone Beam CT of the ICON procedure were used (right).

**Figure 9:**
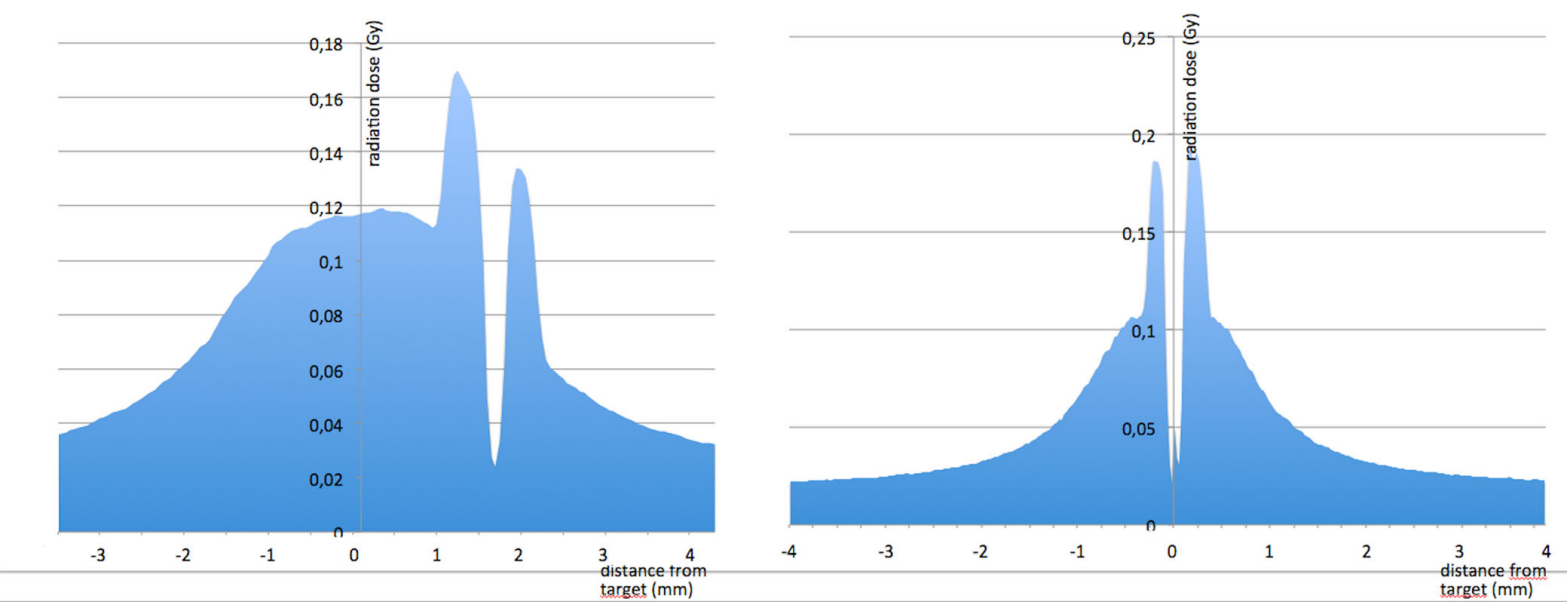
Dose profile analyses of the films using stereotactic coordinates from the CT indicator box (left) and using stereotactic coordinates from the Cone Beam CT of the ICON procedure (right). The stereotactic coordinates of the target were shifted by 2.635 mm on the dose profile of the films using stereotactic coordinates from the CT indicator box. *X*‐axis: measurement of the deviation of the stereotactic X‐coordinate (in mm) from the center of the radiation target. *Y*‐axis: radiation dose (in Gy).

Results of the analysis of gafchromic films of the irradiated target obtained after the correction proposed by the Gamma Knife ICON system had or had not been applied, have confirmed the accuracy of the correction applied. Figure 8 shows gafchromic films after irradiation of the target when the stereotactic coordinates from the CT indicator box were used with a frame not distorted (left figure), when the stereotactic coordinates from the CT indicator box were used with a frame distorted by 3.0 mm (median figure), and when the stereotactic coordinates from the CBCT of the ICON procedure were used with a frame distorted by 3.0 mm (right figure). The target was reached with a high accuracy when the frame was not bent (left figure). With a frame distorted by 3.0 mm, the target was significantly shifted when stereotactic coordinates from the CT indicator box were used (median figure), and was reached with a high accuracy when the stereotactic coordinates from the CBCT of the ICON procedure were used (right figure). Figure 9 shows dose profile analyses of the films using stereotactic coordinates from the CT indicator box (left figure) and those using stereotactic coordinates from the CBCT of the ICON procedure (right figure). The stereotactic coordinates of the target were shifted by 2.635 mm on the dose profile of the films using stereotactic coordinates from the CT indicator box.

## 4 DISCUSSION

Stereotactic procedures require a high level of spatial accuracy. The most classical and efficient way to reach this high level of precision is the use of a rigid stereotactic frame fixed firmly to the patient's head. The frame will give the stereotactic coordinates for surgical or radiosurgical procedures. The stereotactic frame by itself is assumed not to be a source of inaccuracy. Our experiences with Leksell frames G, both clinical and experimental, showed that frames are bendable. We demonstrated that the distortion of the frame is related to the torques applied to the frame during frame fixation to the patient's head. The magnitude, as well as the asymmetry of the torque used, could be a source for frame bending. Other variables not studied in this work, such as the length of fixation posts, the kind of screws used, the angle between screw and skull, etc…, could also influence frame distortion and its potential clinical consequence for stereotactic inaccuracy.

Elekta does not stipulate detailed recommendations for the torque exerted for an adequate frame fixation. In the “Instructions for Use”^3^ provided by Elekta, it is recommended that “do not overtighten” and “avoid excessive force when tightening the fixation screws, otherwise wrong target can be treated”. The explanation for this inaccuracy is that it will be due to bent fixation posts or damaged fixation screws, not to frame bending. This warning has been further addressed by Elekta in a recent Product Bulletin^7^ but the risk of frame distortion is not addressed. On the basis of our study, we suggested that the target inaccuracy induced by overtightening the fixation screws may be due to frame bending.

Maciunas et al.^1^ addressed the problem of application accuracy of stereotactic frames in a well‐conducted study published more than 20 years ago. In this article, the authors show that a potentially significant degree of inaccuracy of stereotactic instruments may occur. Aside from the errors related to imaging, they suggested that the levels of weightbearing used clinically on stereotactic frames, including the Leksell Frame G, could have a pronounced effect on their mechanical accuracy. It was also suggested that frames could have distortion effects when a significant source of torque and weight loading is applied in the frame elements. However, the authors suggested this mechanism only for weight loading of the frame related to positioning of the patient in prone or supine position with the head sharply rotated, they do not mention the torques exerted by application of the frame on the patient's head as a source of frame distortion.

In our clinical study, we tested the variation of the torques exerted by frame application on the patient's head with time. We found no significant difference in the torque measurements taken at the beginning and at the end of the stereotactic procedure. The torque applied by the neurosurgeon on each of the four screws during frame application remained unchanged during the stereotactic procedure, no rebalance between the forces applied to the frame occurs. The analysis of our experience in frame application showed that the torques put on the four screws for head fixation could be different and that significant differences in the torque exerted on the two opposite pairs of screws might lead to a significant distortion of the frame.

Little information on the technique for application of stereotactic head frames could be found in the literature. Safaee et al.^6^ recently published a technical report on this topic. The authors provided recommendations for optimal fixation of the stereotactic head frame based on 25‐years' experience. They routinely used custom torque wrenches to tighten the screws. They recommended securing the frame with torques of 4 inch‐pounds (0.452 Nm). With the same extensive experience in the application of stereotactic head frames, we fixed the Leksell frame with mean torques of 0.348 Nm, a little less than theirs. We did not routinely use torque wrenches for frame fixation instead we applied the stereotactic frame with the wrenches provided by Elekta^®^ (Elekta Instruments AB, Stockholm, Sweden). Safaee et al. did not provide any reference for the assertion that frames could be distorted by overtightening.^6^ Our experiments confirmed that torques greater than 4 inch‐pounds increased the risk of frame distortion, but torques around 4 inch‐pounds could also give rise to significant frame distortion. We then used a box that is not significantly deformable at clinical conditions at the end of the application of the frame to check for frame distortion, and if the frame was distorted, we would modify the conditions of torque on the four screws to correct it. The undeformable box used is a conventional Elekta CT indicator box that was placed on the frame without attachments, and we visually checked if a gap occurred between the box and the frame that represented frame distortion.

The consequences of frame distortion in terms of inaccuracy of targeting in clinical practice represent highly critical information for the quality of stereotactic frame‐based surgical and radiosurgical procedures. The results of our experience using the Target Simulator of Elekta as an experimental model of frame distortion show that distortion of the Leksell frame has a direct and significant impact on the accuracy of targeting for stereotactic procedures using the stereotactic arch. The fixations of the stereotactic arch applied on both sides of the frame are in their turn distorted by frame bending, which will as a consequence influence the accuracy of targeting of the stereotactic instruments. We found a linear relationship between the amount of frame distortion and the extent of deviation from the target.

We performed some experiments to evaluate the accuracy of the stereotactic coordinates issued from CT and MR acquisitions when the Leksell stereotactic frame was subjected to some distortion constraint. We demonstrated that frame distortion will affect significantly the accuracy of the CT‐ and MR‐based stereotactic coordinates. The MR and CT indicator boxes were attached to the frame and when the frame is bent, the imaging series will provide inaccurate stereotactic coordinates. There is a linear relation between the importance of frame distortion and the level of deviation of the stereotactic coordinates issued from imaging acquisition.

For surgical procedures using the Leksell stereotactic arch, the two sources of inaccuracy in targeting will occur: the stereotactic error from imaging acquisition and the stereotactic error from the fixation of the stereotactic arch to the bent frame while for Gamma Knife radiosurgical procedures, only the error from imaging acquisition will occur.

We have shown from our experiment with gafchromic films that the correction proposed by the Gamma Knife ICON system allows correcting efficiently the inaccuracy of targeting induced by frame bending. The stereotactic CBCT of the Gamma Knife ICON apply an accurate correction algorithm. Stereotactic radiosurgery with all devices would probably in the future include systematically a stereotactic 3D imaging acquisition before or even during irradiation to check for inaccuracy of targeting, even when a conventional rigid stereotactic frame is used.

## 5 CONCLUSIONS

The Leksell stereotactic frame G may be subject to distortion induced by the torque exerted by frame attachment to the patient's head. This bending is significantly related to the level of torque applied, and especially to differences of torques applied by the fixation screws. Frame distortion may occur at the levels of torque commonly used clinically. We recommend using torque wrenches for frame fixation, reducing the levels of torque and balancing the torques applied by the four fixations in order to reduce frame distortions. A significant clinical impact of frame distortion on stereotactic procedures or radiosurgical treatments was observed both in surgical and radiosurgical procedures. Image acquisition with the CT‐ or MR‐box and fixation of the stereotactic arch on a distorted frame represented the two sources of inaccuracy in stereotactic targeting. The use of an undeformable box at the end of the application of the frame and use of the CBCT of the Gamma Knife Icon could avoid the consequences of frame distortion as a source of targeting inaccuracy in clinical use.

## CONFLICT OF INTEREST

The authors report no conflict of interest concerning the materials or methods used in this study or the findings specified in this paper.
